# Ultrasound-guided vessel puncture: calling for Pythagoras’ help

**DOI:** 10.1186/s13054-018-2228-1

**Published:** 2018-11-08

**Authors:** Gaël Piton, Gilles Capellier, Hadrien Winiszewski

**Affiliations:** 1 Medical Intensive Care Unit, Besançon University Hospital, Besançon, France; 2 EA3920, Université de Franche Comté, Besançon, France

**Keywords:** Central venous catheter placement, Vessel puncture, Ultrasonography, Technique

Vascular access is routinely performed by critical care physicians and the use of ultrasonography is recommended [1]. However, complications during vessel puncture are not rare, particularly for residents in training with low levels of experience [2]. Reminding about the properties of the isosceles right triangle might be of interest to teach young fellows a secure step by step technique for vessel catheterization.

**Fig. 1:**
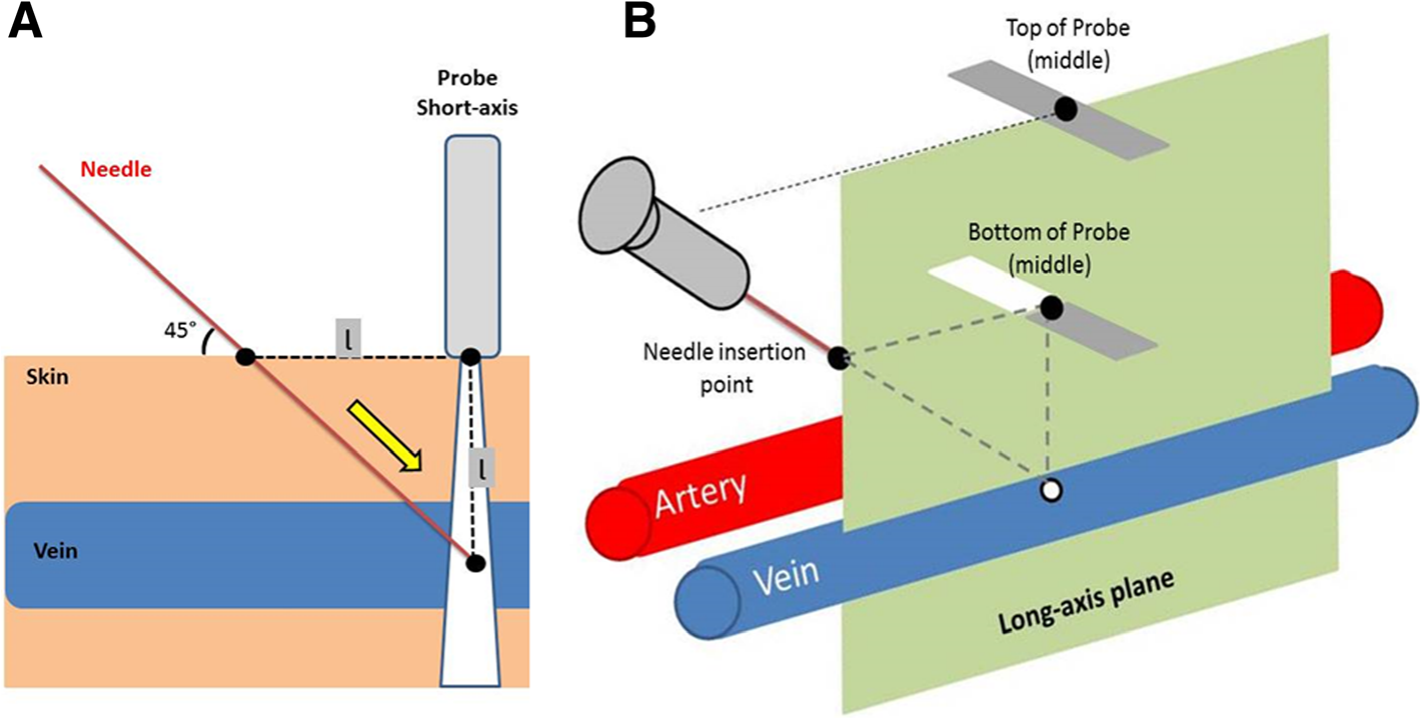
Global overview of ultrasound-guided vessel puncture and use of the isosceles right triangle. **a** Taking a distance between the probe and the needle insertion point equal to the depth of the vessel, a probe perpendicular to the skin, and an angle between the skin and the needle of 45°, the third angle will be automatically positioned into the vein. Three points build an isosceles right triangle: the needle insertion point, the middle of the probe on the skin interface, and the center of the vein under the probe. **b** The long-axis plane, which passes through the length of the vessel, corresponds to the view obtained in the in-plane technique with the vessel in the long-axis. Three points determine the long-axis plane: the needle insertion point, identified by inserting the needle in the skin just above the vessel, the vessel being viewed in the long axis; the middle of the probe on the skin, the vessel being visualized at the center of the ultrasound screen in the short axis; the middle of the probe’s top, the probe being perpendicular to the skin. The operator should attempt to maintain the syringe in the axis of the probe top (*dotted line*)

An isosceles right triangle has two equal legs and has angles of 90°, 45° and 45°. For two equal legs having the length ɭ, the hypotenuse has a length corresponding to ɭ × √2. This last number, also called Pythagoras’ constant, corresponds approximately to 1.4. After a longitudinal scan of the vein run, let’s insert the needle in the skin, just over the vein (Fig. 1a). Let’s position the probe perpendicular to the skin, just over the vein, at a length ɭ of the needle insertion point equal to the depth of the vein under the probe. Three points build an isosceles right triangle: the needle insertion point, the middle of the probe on the skin interface, and the center of the vein under the probe.

The properties of the isosceles right triangle are of interest for the critical care physician teaching fellows for their first venous catheterizations. First, we can determine the needle insertion length required to reach the vein. According to this technique, the needle trajectory from the skin to the vessel corresponds to the hypotenuse of the triangle. For example, if the length ɭ is 2 cm, the needle will have to be inserted 2.8 cm (2 × 1.4) before it reaches the vein. This might help to check whether the needle length is sufficient, in particular for obese patients. Second, we can predict the position of the needle tip in the vein. Taking a distance between the probe and the needle insertion point equal to the depth of the vessel, a probe perpendicular to the skin, and an angle between the skin and the needle of 45°, the third angle will be automatically positioned into the vein. A needle following the hypotenuse of this triangle should reach the center of the vein and make the puncture successful. Third, experts consider that an ideal needle/skin angulation is between 30 and 45° [3]. If the vessel runs parallel to the skin interface, the needle–vessel angulation equals the skin–needle angulation: 45°. This limited angulation of the needle limits the risk of transfixiant puncture but also the risk of kinking of the guidewire during the subsequent dilation procedure.

For ultrasound-guided vessel puncture, in-plane and out-of-plane techniques have advantages and limits. When the long-axis technique is possible and the operator trained, the long-axis technique could be used preferentially. In many situations, however, the short-axis technique is more easily performed, in particular for non-experienced physicians [4]. The technique illustrated here might add advantages of both techniques. First, we can approximate the long-axis plane, which passes through the length of the vessel, by controlling three points in space (Fig. 1b): (1) the needle insertion point, identified by inserting the needle in the skin just above the vessel, the vessel being viewed in the long axis; (2) the middle of the probe on the skin, the vessel being visualized at the center of the ultrasound screen in the short axis; (3) the middle of the probe’s top, the probe being perpendicular to the skin. These three points determine the long-axis plane, which corresponds to the view obtained in the in-plane technique with the vessel in the long-axis. The operator should attempt to maintain the syringe in the axis of the probe top (Fig. 1b, dotted line).The long-axis plane contains the isosceles right triangle. Second, while controlling the long-axis plane by the previously described technique, the use of the short axis view during the vessel puncture allows checking structures lateral to the vessel, in particular arteries and nerves in the case of vein puncture. This is an important point regarding the safety of the procedure.

In conclusion, the basis of Euclidean geometry might be an educational tool for the critical care physician teaching fellows for their first venous catheterizations, giving them a simple step by step procedure.
